# Association of soluble ST2 and infarct location within 12–24 h in STEMI: A cross-sectional study

**DOI:** 10.1016/j.amsu.2021.102844

**Published:** 2021-09-09

**Authors:** Sem David Timothy, Anggoro Budi Hartopo, Vita Yanti Anggraeni, Firdian Makrufardi

**Affiliations:** Faculty of Medicine, Public Health and Nursing, Universitas Gadjah Mada/Dr. Sardjito Hospital, Yogyakarta, Indonesia

**Keywords:** Soluble ST2, Biomarker, Infarct location, ST-segment elevation myocardial infarction (STEMI), Onset of STEMI

## Abstract

**Background:**

ST-Segment Elevation Myocardial Infarction (STEMI) causes the release of soluble ST2 biomarkers at high level on acute phase. However, sST2 has never been used as adjunct biomarker in ESC/AHA guideline for STEMI. Furthermore, the specific onset that sST2 may have role in acute phase of STEMI related with infarct location has not been established. This study aimed to prove the association between serum ST2 levels and infarct location in STEMI.

**Material and methods:**

This study was cross-sectional. STEMI patients with onset of anginal pain 12–24 h were included in study. The exclusion criterias were patients with AMI aside from STEMI and other potential confounders affecting the sST2 level. Serum sST2 was collected on first medical contact when admitted to emergency unit. The patients were grouped into anterior STEMI and non-anterior STEMI. sST2 levels were compared with demographics data, clinical and laboratory variables using Student's t-test. Correlation of sST2 levels was analyzed using Spearman's correlation coefficient.

**Results:**

19 subjects were included in the anterior STEMI and 20 subjects were included in the non-anterior STEMI. We found no difference in sST2 levels between anterior STEMI and non-anterior STEMI (mean ± SD; 729.97 pg/mL ± 147.78 pg/mL vs 606.87 pg/mL ± 147.78 pg/mL, p = 0.119). Onset was correlated with serum sST2 levels in male subjects (r = −0.459, p = 0.012). We found significant difference of mean sST2 between 2 onset groups divided at median (12–18 h vs 19–24 h, Δ mean = 107.75 pg/mL, p-value = 0.021).

**Conclusion:**

sST2 was not associated with infarct location within 12–24 h onset of STEMI. This results suggest that infarct location might not responsible for the elevation of serum sST2 levels in acute phase of STEMI.

## Highlights

• STEMI causes the release of soluble ST2 biomarkers at high level on acute phase.
• sST2 and it association with infarct location within 12–24 h.
• Infarct location might not responsible for the elevation of serum sST2 levels.

## 1 Introduction

ST-segment elevation myocardial infarction (STEMI) is one of the clinical manifestations of acute coronary syndrome in the form of myocardial ischemia characterized by ST segment elevation on the ECG, detection of serum biomarkers and the presence of symptomatic symptoms of persistent chest pain [1,2]. Overall, acute myocardial infarction or ischemic heart disease has been the number one cause of death in the world for more than 15 years [3]. At present, the trend of the prevalence and incidence of acute myocardial infarction tends to increase from year to year even though the STEMI trend tends to decline from year to year [4]. In terms of mortality, STEMI causes higher hospital mortality compared to acute myocardial infarction with non-ST segment elevation (NSTEMI) [5]. In addition, treatment of acute myocardial infarction with percutaneous coronary intervention in patients with STEMI requires a higher cost than STEMI [6,7].

It is well known that acute myocardial infarction that is not treated will lead to ventricular dysfunction and eventually will cause heart failure so that the cost of treatment will be even greater [8]. Therefore, acute myocardial infarction, especially STEMI, is a worldwide health problem in both developed and developing countries [9,10]. As is well known that the occurrence of acute myocardial infarction is caused by the rupture of atherosclerosis plaques in the coronary arteries and eventually causes necrosis of the myocardium. When necrosis of the myocardium occurs, molecularly, the myocardium will secrete various types of biomarkers in the form of proteins. Biomarker that currently often used in the management of acute myocardial infarction is troponin. In fact, many other biomarkers are produced by the myocardium besides troponin. This research on biomarkers is important because each biomarker produced is specific for each cardiovascular case. One of the biomarkers currently being studied is sST2 (soluble Suppression Tumorigenicity-2). sST2 is a dissolved receptor in the blood circulation that binds to its ligand, IL-33 [11].

In the cardiovascular system, sST2 has an important role in cardiovascular disease. Initially, sST2 was reported in a study to play an important role in heart failure as a biomarker of remodeling [12]. In addition, sST2 was also reported to cause the formation of atherosclerosis [13]. Current research in humans only proves that sST2 also plays a role as a predictor of poor outcomes after acute myocardial infarction within 30 days of onset especially in patients with STEMI [14]. Although sST2 has acted as a predictor of poor outcomes after infarction, but the mechanism of induction and regulation of sST2 expression in acute myocardial infarction and sST2 production sites during the process of myocardial infarction in humans are unknown. Until now, there has not been any ideal biomarker to determine the relationship between infarct location and sST2 levels in patients with acute myocardial infarction.

This study aims to determine the relationship of sST2 levels with infarct location in patients with STEMI especially those undergoing revascularization therapy.

## 2 Methods

### 2.1 Patient population

This research was a cross-sectional study. The total subjects were 166 patients with STEMI. The inclusion criteria were both male and female patients aged 35–75 years old with the onset of anginal pain 12–24 h. Exclusion criteria were potential confounders affecting sST2 level including previously known chronic heart failure (New York Heart Association Class > II), chronic kidney disease stage IV-V, hepatic cirrhosis, pneumonia, obstructive lung disease, malignancy, sepsis and acute stroke during observation. The diagnosis STEMI was based on the presence of symptoms consistent with a myocardial infarction, with ST-segment elevation or new left bundle branch block (LBBB) observed during electrocardiography. After diagnosed with STEMI by electrocardiography, the patients were divided into 2 groups. The first group was the anterior infarction group and the second group was non-anterior infarction group. The division of those group was based on ECG findings of STEMI in anterior lead and non-anterior lead. Demographic data, clinical parameters, routine laboratory data, and markers of myocardial necrosis (sST2 and CK-MB) were recorded. The protocol was approved by the Ethics Committee of the Faculty of Medicine, Public Health, and Nursing, Universitas Gadjah Mada, Yogyakarta, Indonesia, approval number KE/FK/494/EC/2016. This study has been reported in line with the STROCSS criteria [14].

### 2.2 Laboratory methods

Samples of venous blood were drawn from antecubital veins in supine position on hospital admission, before coronary angiography and percutaneous coronary intervention (PCI) were performed. Samples were centrifuged for serum isolation. Standard biochemical and hematological blood test were performed using an automated blood cell counter with Sysmex XN-1000, CK-MB was measured using the immunological UV assay method with Cobas c501 (Roche Diagnostics, Switzerland), Troponin I was measured using the enzyme-linked fluorescent assay (ELFA) method with MiniVIDAS (bioMerieux, France). Aliquote of serum was stored at −80’ C until thawed for determination of sST2. sST2 levels were measured using Quantikine Human ST2/IL-33 R Immunoassay (R&D Systems, Minneapolis, MN, USA). The testing was performed by personnel blinded to patient characteristics and clinical outcome.

### 2.3 Clinical outcome

The diagnosis of myocardial infarction specifically, STEMI, was based on electrocardiography (ECG) findings show the elevation of ST segment which counted from J point on minimun two contiguous leads. The condition of ST segment elevation was recorded as elevation ≥0.25 mV (mV) at V2-V3 leads in ≤40 years male, elevation ≥0,3 mV at V2-V3 leads in >40 years male, and elevation ≥0,15 mV at V2-V3 leads in female of all age. In other leads, elevation ≥0,1 mV in male and female of all age. For the infarct location, anterior STEMI includes all leads located in anterolateral, anteroseptal, anteroapical, and anterior-extensive while non-anterior STEMI includes all leads located in lateral, inferior, posterior, and right ventricle. Anterolateral infarction was recorded in lead I, aVL and V5-V6, anteroseptal infarction was recorded in lead V1-V2, anteroapical infarction was recorded in lead V3-V4 and anterior-extensive infarction was recorded in lead I, aVL and V1-V6. Lateral infarction was recorded in lead I and aVL and high lateral was record in lead V5-V6, inferior infarction was recorded in lead II, III and aVF, posterior infarction was recorded in lead V7-V9 (V1R-V2R) and right ventricle infarction was recorded in lead V2R-V4R.

### 2.4 Statistical analysis

Sample size was calculated using formula for the difference in mean of anterior infarction and non-anterior infarction, yielding minimun size of 64 subjects in each group. Standard descriptive statistics were used for analysis. Demographics, clinical, and laboratory variables were described as mean with standard deviation (SD) for continuous variables and number (%) for dichotomous variables. Independent T test, Student's T-test, and χ2 test were used to compare baseline characteristics and clinical outcome with sST2 levels. Spearman correlation coefficient was used to asses the correlation between sST2 and continuous variables. All statistical analysis was performed using IBM SPSS Statistics for Windows, Version 25.0 (IBM Corporation, New York, USA). All p-values are 2-sided with value of <0.05 was considered statistically significant.

## 3 Results

From April 2014 until June 2015, there were total of 166 patients with STEMI and onset of anginal pain ≤24 h admitted to our hospital. As many as 39 patients were eligible as the subjects for this research. The demographic characteristics, clinical parameters, and laboratory results of patients were summarized in Table 1. Baseline characteristics were compared between group of anterior STEMI and non-anterior STEMI. Serum sST2 levels were not normally distributed with mean ± standard deviation (SD) of 702.317 pg/mL ± 148.36 pg/mL (p = 0.04). Infarct locations were significantly associated with hypertension (p = 0.023), systolic blood pressure (p = 0.007), and diastolic blood pressure (p = 0.037). No significant differences were found between infact location and laboratorium parameters including random plasma glucose, creatinine, sST2 levels and leucocyte count.

**Table 1:** Baseline characteristics by infarct location.

Baseline Characteristics	Anterior STEMI (n = 19)	Non-anterior STEMI (n = 20)	p-value
*Demography*			
Age, y	59.11 ± 10.96	58.00 ± 9.72	0.741
Gender			
Male	15 (78.9)	14 (70.0)	0,716
Female	4 (21.1)	16 (30.0)	
Hypertension	16 (84.2)	10 (50.0)	0.023
Diabetes Mellitus	4 (21.1)	8 (40.0)	0.200
Current Smokers	8 (42.1)	8 (40.0)	0.894
Dyslipidemia	3 (15.8)	3 (15.0)	1.000
*Clinical parameters*			
Onset, hour	16.89 ± 4.68	18.30 ± 5.01	0.531
Systolic blood pressure, mmHg	146.05 ± 30.84	121.70 ± 22.23	0.007
Diastolic blood pressure, mmHg	85.68 ± 16.68	75.20 ± 13.52	0.037
Heart rate, beats/minute	81.58 ± 15.59	71.30 ± 22.32	0.073
*Laboratorium results*			
Random plasma glucose, mg/dL	168.57 ± 63.73	210.95 ± 139.85	0.922
Creatinine, mg/dL	1.24 ± 0.39	1.32 ± 0.64	0.757
sST2, pg/mL	729.97 ± 147.78	606.87 ± 147.78	0.119
Leucocyte count, 10^3^/mm^3^	12.44 ± 2.67	14.13 ± 4.40	0.158
*Medical Intervention*			
Primary PCI	10 (52.6)	7 (35.0)	0.267
Fibrinolytic	3 (15.8)	5 (25.0)	0.695

PCI: percutaneous coronary intervention.

### 3.1 Correlation of sST2 levels with baseline characteristics

sST2 levels were found to have statistically significant negative and weak correlation with onset in male subjects (r = −0.372, p = 0.020) while in female subject, sST2 levels were found to have statistically significant positive and weak correlation random blood glucose (r = 0.657, p = 0.039) as presented in Table 2.

**Table 2:** Correlation of sST2 levels with baseline characteristics

Baseline Characteristics	Spearman's *r*	p-value
Age	0.041	0.804
Onset	−0.372	0.020
Male	−0.459	0.012
Female	0.055	0.880
Systolic blood pressure	0.245	0.133
Diastolic blood pressure	0.203	0.216
Heart rate	−0.145	0.380
Leucocyte count	−0.211	0.198
Creatinine	0.126	0.445
Random blood glucose	0.259	0.112
Male	0.101	0.603
Female	0.657	0.039

## 4 Discussion

**Fig. 1:**
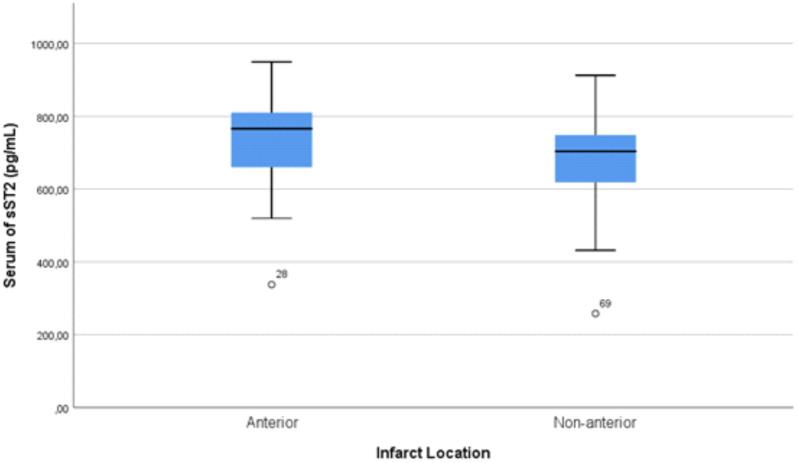
Serum sST2 levels of STEMI patients within range of 12–24 h since onset measured on first medical contact and categorized based on the infarct location (p-value = 0.119).

**Fig. 2:**
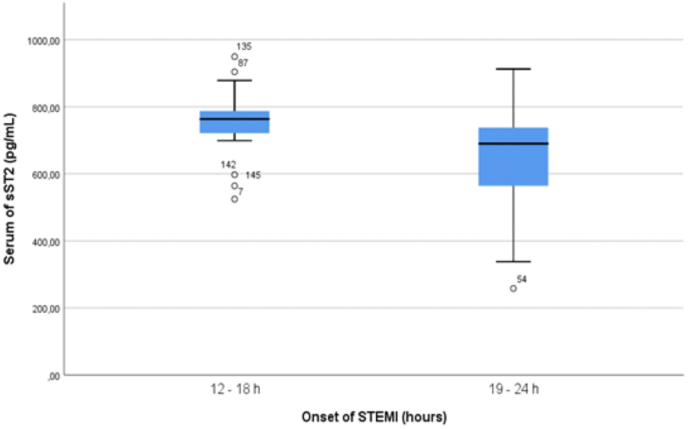
Serum sST2 levels of patients with STEMI, measure on hospital admission and categorized based on the median (18 h) onset of anginal pain (p-value = 0.021).

We found in present study that sST2 levels have no statistically significant differences with infarct location both anterior infarction and non-anterior infarction within range 12–24 h since onset of anginal pain (Fig. 1). There are two studies involving patients with acute myocardial infarction that supported this study. In the first study with randomized, double-blinded, placebo-controlled clinical trial [16], the researchers found that there no significant difference between serum sST2 levels and sites of infarction, in their case, the sites of infarction were anterior infarction, anterolateral, inferior inferolateral and lateral. A study which gathered 39 patients and 42 healthy individuals [17] also found the same results but with different infarct locations/sites. The infarction locations were anterior, anterolateral, and anteroseptal. Both studies were complementing to each other in terms of infarct locations. The insignificant differences might can be explained by the first study mentioned above. The first study [16] shows that although serum sST2 levels did not correlated with infarct location but they found that serum sST2 levels correlated with baseline infarct volume, endocardial extent of infarction and was higher in patients with greater infarct transmurality. These suggest that patients with STEMI, which causes transmural infarction, have a higher serum sST2 level. Anatomically, the anterior location reflects a wider and thicker left ventricle than non-anterior location. The subendocardial infarction in this anterior location in patients with STEMI causes a higher release of serum sST2 which is proved in our study. In our study, the mean serum sST2 level of anterior location was 729.97 pg/mL while the mean serum sST2 level of non-anterior location was 606.87 pg/mL. The mean difference was equal to 123.1 pg/mL and it was not statistically significant (p-value = 0.119). We predicted that there are some molecular mechanism that cause this difference. This lead to another premise that might be a mechanism in which myocardial strain in anterior location may be stronger than the non-anterior location that causes reduction in straining of ventricle in non-anterior location or there might be other non-myocardial sources of serum sST2 production other than cardiac muscle as shown in previous study such as endothelial artery, aortic artery and coronary artery [[18], [19], [20]]. In this study, we also used onset within range of 12–24 h because it was known from the previous studies with randomized, open-label method that the peak concentration of sST2 was achieved by most patients at 12 h [15] and some patients at 24 h [21]. The other premise we used the onset range of 12–24 h is that we expect that serum sST2 would only be released at a certain specific period of time other than just the location of infarction. This is supported by a finding in our study is that sST2 levels significantly correlated negatively and weakly with onset in subjects with STEMI without or with adjusting to male sex type (Table 2). In present study, 24 h is upper limit of our onset. This means that as the onset of STEMI increases, the serum sST2 levels decreases and vice versa. We also depicted the mean difference of serum sST2 levels based on our median onset of STEMI and found that mean difference was statistically significant between two groups (12–18 h vs 19–24 h, Δ mean = 107.75 pg/mL, p-value = 0.021). Based on Fig. 2, we also can conclude that higher sST2 levels were produced within specific period range of 12–18 h onset of STEMI rather than within range of 18–24 h onset of STEMI. This finding are similar with previous studies that show a decrease of serum sST2 levels overtime as it reaches 24 h and above [15,16,22]. We predicted that this was caused by acute inflammation that progressed into chronic inflammation causing sST2 concentration gradually achieved its peak within 12–24 h.

We also found in our study is that sST2 levels significantly correlated positively and weakly with random blood glucose in subjects with STEMI after adjusting to female sex type (Table 2). Previous studies showed that serum sST2 did correlate with blood glucose in subjects with STEMI [23] and NSTEMI [24] without adjusting to sex type. We still do not know how glucose correlated with sST2 in female subjects with STEMI but we predict that hormone especially estrogen plays important role regulating insulin between male and female as stated in review study [25].

**Fig. 3:**
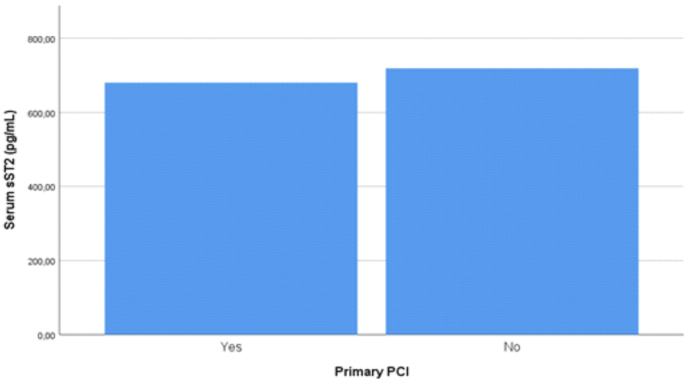
Serum sST2 levels of patients with STEMI, measure on hospital admission and categorized based on primary PCI procedure (p-value = 0.561).

**Fig. 4:**
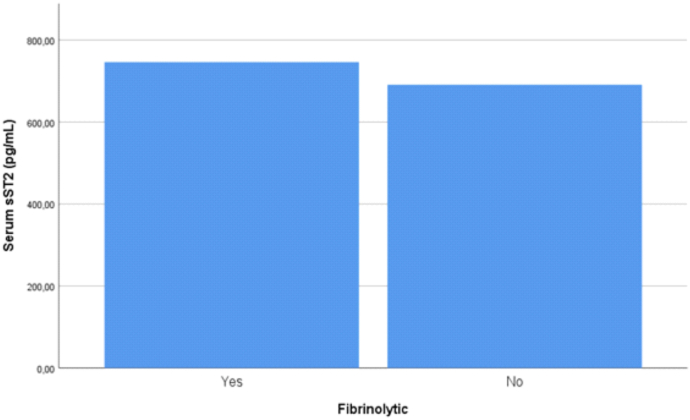
Serum sST2 levels of patients with STEMI, measure on hospital admission and categorized based on fibrinolytic procedure (p-value = 0.356).

Our study also did not differ which patient had undergone revascularization procedure such as primary percutaneous coronary intervention and fibrinolytic but we found respectively that there were no statistically significant between PCI procedure (p-value = 0.267) and fibrinolytic therapy (p-value = 0.695) in both group infarct location and we measured the mean difference of serum sST2 level in both primary PCI and fibrinolytic. We found that mean difference of sST2 serum was lower in patient treated in primary PCI than those who did not treated with primary PCI (680.48 pg/mL vs 719.19 pg/mL, p-value = 0.561, Fig. 3) and we also found that mean sST2 serum was higher in patient treated with fibrinolytic than those who did not receive fibrinolytic (746.14 pg/mL vs 691.00 pg/mL, p-value = 0.356, Fig. 4) although both results were not statistically significant. In previous studies with 301 patients, it was explained that fibrinolytic therapy was less effective in restoring coronary blood flow and reducing infarct size thus causing more cellular injury and increase risk of re-infarction which subsequently release more serum sST2 than primary PCI [26].

As mentioned before, sST2 was not only produced by myocardial but also the macrovascular and microvascular of the heart. In fact, the previous studies also shows genetic expression of ST2 mRNA were vastly expressed in those vasculature than the cardiac myocyte, cardiac fibroblast and vascular smooth muscle resulting the higher expression of serum sST2 [[18], [19], [20]]. Considering this non-myocardial production of serum sST2, sST2 may not be useful for diagnostic purpose in the setting of STEMI because functionally, the baseline levels of sST2 may not reflect the true cardiac condition of the patient in acute condition [26].

Given the peak of serum sST2 levels mostly above 12–24 h of STEMI onset, sST2 may not be useful in acute condition but probably more valuable in chronic condition which is above 12 h of STEMI onset. This rationale lead to 2017 ESC STEMI guidelines that recommended PCI should be done within 2 h of STEMI onset and fibrinolytic should be done before 12 h of STEMI onset [1]. These conditions are acute condition whereas serum sST2 has not achieve the peak condition. Previous study with cohort design even showed that measured sST2 level within 24 h (early after infarction) with point of care test could provide valuable prognostic information for adverse cardiac events [27]. By assessing this condition, sST2 may be more suitable as prognostic biomarker in patients with STEMI alongside with high sensitivity troponin to stratify the risk of mortality in STEMI patients. In this way, sST2 levels provided incremental value as an adjunct biomarker as it has been validated as prognostic biomarker alongside high sensitivity troponin (Hs-troponin) in either 2017 ESC guidelines of STEMI [1] or 2013 AHA guidelines of STEMI [2].

Potential limitation of the study merit consideration, foremostly the limited number of patients. Several exclusion criteria that were used in the patient population could not be controlled. These might restrict the applicability of the research results into STEMI population. Furthermore, there were no controlled subject in our study and the patients were not differed between treated or not treated with reperfusion therapy.

Future studies should consider more stringent criteria of exclusion and controlled subject knowing that sST2 is well-versatiled biomarker that can be used in multi-studies and that can be affected by multiple conditions. Comparing to controlled subjects can provide more robust information about the general population. Furthermore, use of standard biomarker such troponin along with sST2 is necessary to analyze the complementary roles and give incremental value for prognostic-wise of mortality in patients with STEMI.

## 5 Conclusion

Elevated levels of sST2 are not significantly associated with infarct location within range of 12–24 h since onset of STEMI. This results suggest sST2 levels may only be produced at high concentration in a certain period of time, in this study within range of 12–18 h of onset and high sST2 levels on admission was not affected by type of infarct location. Therefore, future studies should assess value of repeated sST2 levels measurement in chronic phase of STEMI for better understanding of underlying pathophysiology process of sST2 secretion and as prognostic biomarker for patients outcome.

## Consent statement

Written informed consent was obtained from all of the patients for publication of study and accompanying images. A copy of the written consent is available for review by the Editor-in-Chief of this journal on request.

## Funding sources

The authors declare that this study had no funding source.

## Provenance and peer review

Not commissioned, externally peer-reviewed.

## Declaration of competing interest

The author(s) declared no potential conflicts of interest with respect to the research, authorship, and/or publication of this article.
